# Tumor Necrosis Factor Receptor Superfamily Member 21 Induces Endothelial-Mesenchymal Transition in Coronary Artery Endothelium of Type 2 Diabetes Mellitus

**DOI:** 10.3390/biomedicines10061282

**Published:** 2022-05-30

**Authors:** Po-Chao Hsu, Jiun-Chi Huang, Wei-Chung Tsai, Wei-Wen Hung, Wei-An Chang, Ling-Yu Wu, Chao-Yuan Chang, Yi-Chun Tsai, Ya-Ling Hsu

**Affiliations:** 1 Division of Cardiology, Kaohsiung Medical University Hospital, Kaohsiung Medical University, Kaohsiung 807, Taiwan; 920255@gap.kmu.edu.tw (P.-C.H.); k920265@kmu.edu.tw (W.-C.T.); 2 School of Medicine, College of Medicine, Kaohsiung Medical University, Kaohsiung 807, Taiwan; jchuang@kmu.edu.tw (J.-C.H.); 960215kmuh@gmail.com (W.-A.C.); 3 Division of Nephrology, Kaohsiung Medical University Hospital, Kaohsiung Medical University, Kaohsiung 807, Taiwan; 4 Division of Endocrinology and Metabolism, Kaohsiung Medical University Hospital, Kaohsiung Medical University, Kaohsiung 807, Taiwan; 900084@mail.kmuh.org.tw; 5 Division of Pulmonary and Critical Care Medicine, Kaohsiung Medical University Hospital, Medical University, Kaohsiung 807, Taiwan; 6 Graduate Institute of Clinical Medicine, College of Medicine, Kaohsiung Medical University, Kaohsiung 807, Taiwan; r060072@kmu.edu.tw; 7 Department of Anatomy, Kaohsiung Medical University, Kaohsiung 807, Taiwan; chaoyuah@kmu.edu.tw; 8 Division of General Medicine, Kaohsiung Medical University Hospital, Kaohsiung Medical University, Kaohsiung 807, Taiwan; 9 Liquid Biopsy and Cohort Research, Kaohsiung Medical University, Kaohsiung 807, Taiwan; 10 Drug Development and Value Creation Research Center, Kaohsiung Medical University, Kaohsiung 807, Taiwan; 11 Graduate Institute of Medicine, College of Medicine, Kaohsiung Medical University, Kaohsiung 807, Taiwan

**Keywords:** TNFRSF21, endothelial–mesenchymal transition, permeability, coronary artery endothelial cell, diabetes mellitus

## Abstract

Diabetes mellitus (DM) is an increasing threat to human health and regarded as an important public issue. Coronary artery disease is one of the main causes of death in type 2 DM patients. However, the effect of hyperglycemia on coronary artery endothelial cells (CAECs) and the pathophysiologic mechanisms are still not well-explored. This study aims to explore the signal pathway and novel biomarkers of injury of CAECs in DM in understanding the microenvironment changes and mechanisms of diabetic heart disease. Next-generation sequence (NGS) and bioinformatics analysis to analyze the CAECs of one type 2 DM patient and one normal individual was performed, and it was found that tumor necrosis factor receptor superfamily member 21 (TNFRSF21) was a soluble factor in circulating system. Further experiments confirmed that advanced glycation end products (AGEs), the metabolite derived by hyperglycemia, increased the expression of TNFRSF21 in CAECs. TNFRSF21 induced endothelial–mesenchymal transition (EndoMT) in CAECs, resulting in increased permeability of CAECs. In addition, levels of serum TNFRSF21 were higher in type 2 DM patients with left ventricular hypertrophy (LVH) than those without LVH. Serum TNFRSF21 levels were also positively correlated with the LV mass index and negatively with LV systolic function. Serum TNFRSF21 levels were associated with changes in cardiac structure and function in patients with type 2 DM. In conclusion, TNFRSF21 plays a pathogenic role in heart disease of type 2 DM, and can be used as a biomarker of the impairment of cardiac structure and function in type 2 DM patients.

## 1. Introduction

Diabetes mellitus (DM) is a growing global public health problem [1], as it is a chronic metabolic disease characterized by elevated blood sugar, which leads to serious damage to multiple organs or tissues such as heart, kidney, vessels, eye, or nerve [2]. Type 2 DM is the most common type of DM and is known as adult-onset diabetes [3]. The prevalence of type 2 DM has risen dramatically in most countries in the past decades, causing multiple morbidities and high mortality. Type 2 DM has a complex pathophysiology composed of several underlying defects leading to impaired glucose homeostasis and the development of microvascular and macrovascular complications [4]. Retinopathy, neuropathy, and nephropathy are so-called microvascular complications, and coronary artery disease (CAD), cerebrovascular disease, and peripheral vascular disease belong to macrovascular complications. Of all patients with type 2 DM, 32.2% had cardiovascular disease (CVD), 29.1% had atherosclerosis, 21.2% had coronary heart disease, 14.9% heart failure, 14.6% angina, 10.0% myocardial infarction and 7.6% stroke [4]. Compared to normal individuals, type 2 DM patients have higher prevalence rate of CVD, accounting for approximately half of all deaths [5,6]. Because CAD may further lead to heart failure, arrhythmia, and even myocardial infarction [5], how to prevent DM related macrovascular disease is extremely important to decrease the possible morbidities and mortalities. In addition to the effect of oral antidiabetic drugs on prevention of macrovascular diseases [7,8], multifactorial interventions, such as intensive blood pressure and lipid control, have also shown great benefits in prevention of DM-related macrovascular complications [9].

Endothelial cell is the cell lying on the inner surface of blood vessels and lymphatic vessels, and its main function is to maintain perfusion and immunity in microenvironment within the tissue by regulating the permeability of blood vessels and allowing immune cells to penetrate [10]. Hyperglycemia is a risk factor of causing vascular damage. High glucose (HG) exerts a negative effect on both coronary circulation and myocardial tissue by several pathophysiological mechanisms, which promote activation of inflammation, cellular damage, endothelial dysfunction, coagulation cascade and increased platelet aggregation [11,12]. HG promotes synthesis of platelet-derived growth factors in endothelial cells, leading to accumulation of extracellular matrix, resulting in abnormal endothelial cell function [13,14]. In addition, advanced glycation end products (AGEs), a metabolite of hyperglycemia, have been found to reduce nitric oxide synthase through activating p38 and ERK1/2, leading to coronary vascular endothelial cell damage [15]. Furthermore, abnormal endothelial cell function lead to the impairment of heart function and promote heart disease in diabetic patients [16]. If novel pathophysiological mechanisms or signal transmission pathways induced by hyperglycemia can be discovered, they will assist in early detection and treatment of CVD caused by DM.

Recent research has pointed that endothelial–mesenchymal transition (EndoMT) of endothelial cells causes diabetic heart disease in DM patients [17,18]. The characteristic of EndoMT is the transformation of endothelial cell phenotypes, including loss of cell–cell junctions, down-regulation of protein expression resulting in loss of cell attachment, cytoskeleton reorganization, and increased extracellular matrix. EndoMT is involved in organ fibrosis through both transforming growth factor beta (TGF-β)-dependent and TGF-β-independent signaling pathways [17]. Several pathogenic factors, such as inflammatory cytokines, metabolic abnormalities, and AGEs, induce EndoMT in endothelial cells through negative regulation of sirtuin 1 or positive regulation of TGF-β signaling pathway [18,19]. According to the above findings, preventing or reducing EndoMT in endothelial cells has been the potential strategy for the treatment of CVD in DM.

Therefore, the aim of this project is to explore the signal pathway of EndoMT in the cardiovascular system, and furthermore, develop new treatment strategies and establish novel prognostic markers in CVD of type 2 DM.

## 2. Materials and Methods

### 2.1. Cell Lines and Cell Cultures

Primary coronary artery endothelial cells (CAECs) of a DM patient (Cat CC-2922) and a normal individual (Cat CC-2585) (Lonza, Walkersville, MD, USA) were cultured in Medium-2 BulletkitTM (Cat CC-3162). Human CAECs (Sciencell, Cat 6020) were cultured in Endothelial Cell Medium (ECM, Cat 1001) plus 5% Fetal Bovine Serum (FBS). The characteristics of cells were identified by observations of their morphology using light microscopy (Nikon ECLIPSE TE20000-S, Nikon, Tokyo, Japan). Cells were treated with normal glucose (NG, 5.5 mM), HG (25 mM), bovine serum albumin (BSA, 300 μg/mL, Sigma-Aldrich, St. Louis, MA, USA), AGE-BSA (300 μg/mL, Sigma-Aldrich, St. Louis, MA, USA), and tumor necrosis factor receptor superfamily member 21 (TNFRSF21 (DR6/Fc), 10 ng/mL, R & D system, Minneapolis, MN, USA) for the indicated times.

### 2.2. RNA Sequencing and Bioinformatics Analysis

Transcriptome of human diabetic primary CAECs and normal primary CAECs were collected and profiled by next generation sequencing (NGS). Total RNA from harvested cells was extracted using Trizol**^®^** Reagent (Invitrogen, Carlsbad, CA, USA). The quality of extracted RNA was analyzed using an ND-1000 spectrophotometer (Nanodrop Technology, Wilmington, DE, USA) and was confirmed by RNA integrity number by Agilent Bioanalyzer (Agilent Technology, Santa Clara, CA, USA). Samples were readied for RNA preparation and sequencing analysis by Welgene Biotechnology Company (Taipei, Taiwan) as our previous study [20]. The differentially expressed gene between diabetic and normal CAECs was set at fold change > 2.0, fragments per kilobase of transcript per million (FPKM) > 0.3 for mRNA. The updated Database for Annotation, Visualization and Integrated Discovery (DAVID) Bioinformatics Resources (https://david.ncifcrf.gov/, accessed on 1 Febuary 2022) was used to analyze large gene lists. Ingenuity Pathway Analysis (IPA) software (Ingenuity systems, Redwood City, CA, USA) provided “Core Analysis” and “Tox list” for the bio-function of genes/proteins. 

### 2.3. Real-Time Quantitative Reverse Transcription PCR (qRT-PCR)

After total RNA extraction of cells, oligo (dT) primer and reverse transcriptase (RT; Takara, Shiga, Japan) were used to prepare the cDNA. Quantitative RNA was analyzed by SYBR Green system on the QuantStudio3 (Thermo Fisher, Waltham, MA, USA). PCR reaction was performed with the following temperature profile (95 °C for 10 min, followed by 40 cycles at 95 °C for 15 s and 60 °C for 1 min). Relative expression levels of the mRNA in cells were normalized to interval control GAPDH. Relative expression was presented by the 2^−ΔΔCt^ method. The primers (TNFRSF21, TNFSF4, cadherin 11 (CDH11), protocadherin 7 (PCDH7), protocadherin 10 (PCDH10) and GAPDH) used are listed in Table S1.

### 2.4. Human Study Participants

One hundred and thirty type 2 DM patients were enrolled. Type 2 DM was defined as a medical history of DM or the use of anti-diabetes agents. Demographic and medical data were obtained from medical records and interviews with study participants. Blood pressure was measured after the patients had seated and rested for 5 min. The mean of three consecutive blood pressure measured by a single calibrated device at 5 min intervals was used for analysis. Body mass index (BMI) was calculated as body weight/body height squared (kg/m^2^). The study was approved by the Institutional Review Board of the Kaohsiung Medical University Hospital (KMUHIRB-G(I)20160036 and KMUHIRB-G(I)20170037).

### 2.5. Measurement of Cardiac Structure and Function 

Cardiac structure and function were evaluated as previously reported [21]. In brief, experienced cardiologists blinded to the clinical characteristics and laboratory data performed echocardiographic examinations on the same provided blood samples using a VIVID 7 ultrasound system (General Electric Medical Systems, Horten, Norway). M-mode and two-dimensional images were obtained from standard views. The echocardiographic parameters such as left ventricular internal diameter, left ventricular posterior wall thickness, and interventricular septal wall thickness in diastole and systole, left atrial diameter (LAD), peak early transmitral filling wave velocity (E), and peak late transmitral filling wave velocity (A) were obtained. Systolic function of left ventricle was assessed using left ventricular fractional shortening (LVFS) and left ventricular ejection fraction (LVEF), while left ventricular mass (LVM) was calculated using Devereux-modified method [22]. Left ventricular mass index (LVMI) was calculated as LVM divided by body surface area, while left ventricular hypertrophy (LVH) was defined according to the 2007 European Society of Hypertension/ European Society of Cardiology guidelines [23]. Diastolic dysfunction was defined as an E/A ratio < 1. 

### 2.6. Quantification of Serum TNFRSF21 and TNFSF4 in Supernatant of HCAECs and Serum of Patients

Supernatants of HCAECs treated with NG and HG for 48 h were collected. Study participants were asked to fast for at least 12 h before the collection of blood samples, which were then aliquoted and stored in a −80 °C freezer. TNFRSF21 (Cat DY144) and TNFSF4 (Cat DY1054-5) levels of supernatant of CAECs and human serum were measured using enzyme-linked immunosorbent assay (ELISA) (R & D system, Minneapolis, MN, USA).

### 2.7. Western Blot Analysis

Radio-Immunoprecipitation Assay Lysis buffer (EMD Millipore, Burlington, MA, USA) was used to lyse HCAECs. Equal amounts of protein were subjected to 9% SDS-PAGE for electrophoresis, followed by transfer onto a polyvinylidene difluoride membrane. After blocking with 5% blocking buffer, the membrane was immunoblotted with primary antibodies overnight at 4 °C. The membrane was washed with tris-buffered saline/Tween-20 (0.2%) and then incubated with a horseradish peroxidase (HRP)-conjugated secondary antibodies. The corresponding bands were examined by a chemiluminescent HRP substrate kit (EMD Millipore, Burlington, MA, USA), and chemiluminescence signals were assessed using Proteinsimple + Fluorchem Q (Alpha Innotech, San Leandro, CA, USA). Antibodies against N-cadherin (Cat 610921, 1:2000), and vimentin (Cat 550513, 1:2000), E-cadherin (Cat 610182, 1:2000) from BD Biosciences (Franklin Lakes, NJ, USA), alpha smooth muscle actin antibody (α-SMA, 1:1000, Cat ab5694, Abcam) and GAPDH (Cat mab374, 1:3000) from EMD Millipore (Burlington, MA, USA) and endothelial Nitric Oxide Synthase (eNOS, Cat 32027, 1:2000) and VE-cadherin (Cat 32027, 1:2000) from Cell Signaling (Danvers, MA, USA) were used. The densitometry of the bands was analyzed using Image J software version (https://imagej.net/WelcomeUSA, accessed on 15 January 2022).

### 2.8. Permeability Analysis of HCAECs

Transendothelial permeability assay was performed using In Vitro Vascular Permeability Assay kit (EMD Millipore, Burlington, MA, USA). HCAECs grown to confluence on collagen-coated inserts were exposed to NG, HG, BSA, BSA-AGE, normal control (NC) and TNFRSF21 (DR6/Fc) for 48 h. After treatment, FITC-labelled dextran was added to the top of the cell monolayer for 2 h, and then FITC-dextran across the HCAECs monolayer to the bottom wells was measured by relative fluorescence excitation at 485 nm and emission at 530 nm using a fluorescence plate reader.

### 2.9. Statistical Analysis

Continuous variables were expressed as mean ± standard error of the mean (S.E.M.) or median (25th, 75th percentile), as appropriate, with categorical variables expressed as percentages. Differences in categorical variables were tested using the Chi-square test, while significance of differences in continuous variables between the groups was tested using Student’s *t*-test. The association among continuous variables was examined by Spearman correlation. Statistical analyses were conducted using SPSS version 22.0 for Windows (SPSS Inc., Chicago, IL, USA) and Graph Pad Prism 9.2.0 (GraphPad Software Inc., San Diego, CA, USA). Statistical significance was set at a two-sided *p*-value of <0.05.

## 3. Results

### 3.1. Identification of Differentially Expressed Genes Associated with CAEC Injury of Type 2 DM Patients and Normal Individuals

**Figure 1:**
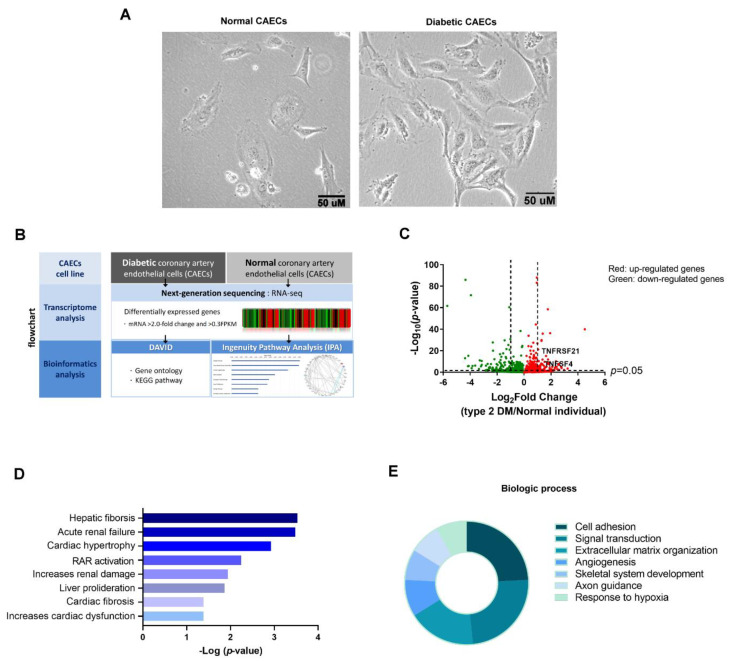
Identification of differentially expressed genes correlated with CAEC injury of the type 2 DM patient and the normal individual. (**A**) The morphological changes in normal and diabetic CAECs. (**B**) Flowchart of identification of potential genes correlated with HCAECs injury in type 2 DM. (**C**) Display of differential expression patterns of normal and diabetic CAECs from deep RNA sequencing by volcano plot. The 112 differentially expressed genes between diabetic and normal CAECs were analyzed using (**D**) Tox list of IPA analysis and (**E**) biologic process in DAVID database. The top seven categories of these dysregulated genes in diabetic CAECs are displayed in a pie chart. The pie chart indicates the-Log10 (false discovery rate, FDR) of each term, and the numbers that are shown at the outside of each pie segment indicates the number of genes involved in each term.

Normal CAECs had more cuboidal epithelial structures and diabetic CAECs had more elongated mesenchymal shape (Figure 1A). To investigate genes that were potentially correlated with HCAEC injury in type 2 DM, we collected RNA samples from CAECs of one normal individual and one DM patient and used NGS to profile transcriptomes, and then candidate regulators were analyzed using bioinformatics tools (Figure 1B). The differentially expressed genes in normal and diabetic CAECs is shown in Figure 1C. Differentially expressed protein-coding genes between normal and diabetic CAECs were filtered with >2.0-fold-change, with a threshold setting of >0.3 FPKM. Finally, 48 mRNAs had up-expression and 64 mRNAs had down-expression in diabetic CAECs compared with normal CAECs. 

To understand the biological functions of the 91 differentially expressed genes in diabetic CAECs, these genes were input into IPA and DAVID database for core analysis or enrichment analysis. Tox lists of IPA analysis revealed these mRNAs might participate in cardiac injury, including cardiac hypertrophy, cardiac fibrosis, and increased cardiac dysfunction (Figure 1D). DAVID database indicated these mRNAs were associated with extracellular matrix organization and cell adhesion in biologic process (Figure 1E). These results support that dysfunction of extracellular matrix and cell adhesion is involved in CAECs of type 2 DM. Based on the results of NGS with bioinformatics analysis, we extensively used qRT-PCR and protein detection techniques to validate these target genes.

### 3.2. AGEs Increased TNFRSF21 Expression in CAECs of Type 2 DM

**Figure 2:**
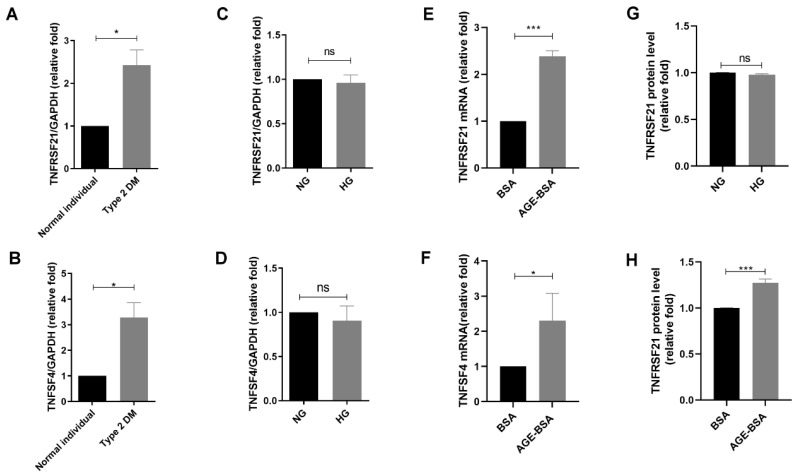
Increased TNFRSF21 and TNFSF4 expression in HCAECs in type 2 DM. (**A**,**B**) TNFRSF21 and TNFSF4 mRNA expression in CAECs from a normal individual and type 2 DM patient (*n* = 3). (**C**,**D**) TNFRSF21 and TNFSF4 mRNA expression in HCAECs treated with NG (5.5 mM) or HG (25 mM) for 24 h (*n* = 3). (**E**,**F**) TNFRSF21 and TNFSF4 mRNA expression in HCAECs treated with BSA (300 μg/mL) or AGE-BSA (300 μg/mL) for 24 h (*n* = 3). TNFRSF21 and TNFSF4 mRNA levels were assessed by quantitative real-time polymerase chain reaction (qRT-PCR). (**G**,**H**) TNFRSF21 protein expression in the supernatant of HCAECs treated with NG, HG, BSA, and AGE-BSA for 48 h using enzyme-linked immunosorbent assay. The bar graph represents the mean ± S.E.M. * *p* < 0.05, *** *p* < 0.001 by Student’s *t*-test. ns: non-significance.

Among 38 up-regulated mRNAs in diabetic CAECs, TNFRSF21 and TNFSF4 were soluble factors that might transmit biologic signals by circulating system (Table 1). We examined which factors affected TNFRSF21 and TNFSF4 expression in CAECs. Increased expression of TNFRSF21 and TNFSF4 mRNA was seen in diabetic CAECs compared to normal CAECs (Figure 2A,B); however, HG did not affect either TNFRSF21 or TNFSF4 expression at mRNA level in HCAECs (Figure 2C,D). In contrast, TNFRSF21 and TNFSF4 mRNA levels increased in HCAECs that were treated with AGE-BSA for 24 h (Figure 2 E,F). We further measured TNFRSF21 and TNFSF4 at protein level in supernatant of HCAECs treated with BSA and AGE-BSA for 48 h, and found AGE-BSA, not HG, increased TNFRSF21 levels in supernatant of HCAECs (Figure 2 G,H); however, TNFSF4 expression in the supernatant of HCAECs was undetectable by ELISA-based methods. These findings support that AGEs, but not HG, enhanced TNFRSF21 expression and secretion in CAECs. 

### 3.3. Elevated Serum TNFRSF21 Levels Correlated with Impaired Cardiac Structure and Function in Patients with Type 2 DM

**Figure 3:**
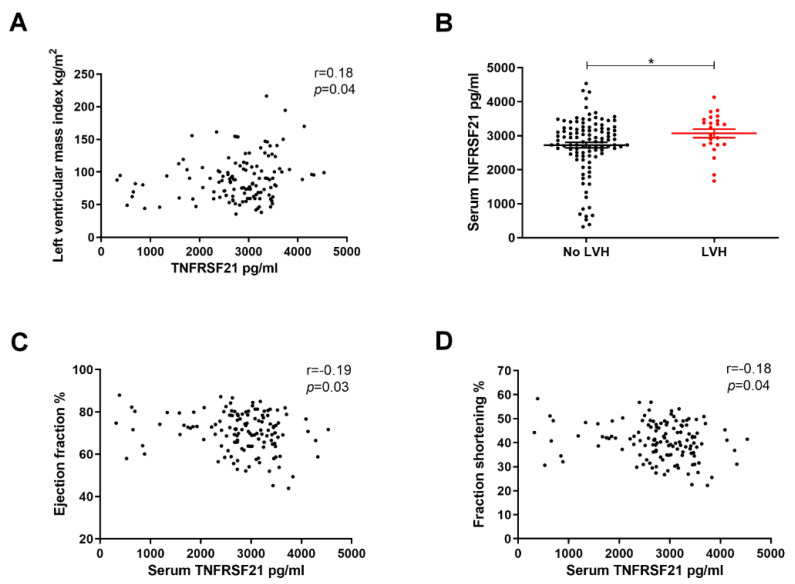
Elevated serum TNFRSF21 levels correlated with impaired cardiac structure and function in type 2 DM (**A**) The correlation between left ventricular mass index and serum TNFRSF21 level (*n* = 130). (**B**) Serum TNFRSF21 level in type 2 DM patients with LVH and those without LVH. (**C**,**D**) The association serum TNFRSF21 level with left ventricular ejection fraction and fraction shortening. Serum TNFRSF21 level was measured using enzyme-linked immunosorbent assay. The bar graph represents the mean ± S.E.M. * *p* < 0.05 by Student *t*-test, and *p*-value of correlation was analyzed by Spearman analysis.

Since TNFRSF21 and TNFSF4 mRNA levels were elevated in CAECs of type 2 DM, we further examined the impact of serum TNFRSF21 and TNFSF4 levels on cardiac structure and function of type 2 DM patients. One hundred and thirty type 2 DM patients were enrolled, and were stratified by the existence of LVH (Table 2). Twenty-three type 2 DM patients had LVH. No significant difference in age and sex distribution and sugar control between the two groups was found. The mean of serum TNFRSF21 level was 2789.4 ± 807.5 pg/mL in all type 2 DM patients, and positive correlation between serum TNFRSF21 and LVMI was found (Figure 3A). Type 2 DM patients with LVH had higher levels of serum TNFRSF21 compared with those without LVH (Figure 3B). In addition, high serum TNFRSF21 level was associated with low left ventricular ejection fraction (Figure 3C) and left ventricular fraction shortening (Figure 3D). There was no significant relationship between serum TNFRSF21 level and diastolic dysfunction (data not shown). In addition, serum TNFSF4 level was also assessed, but the level was too low to be determined in the serum of type 2 DM patients. Thus, TNFRSF21, but not TNFSF4, was found to have potential role in impaired cardiac structure and function in type 2 DM.

### 3.4. TNFRSF21 Promoted EndoMT in CAECs of Type 2 DM

**Figure 4:**
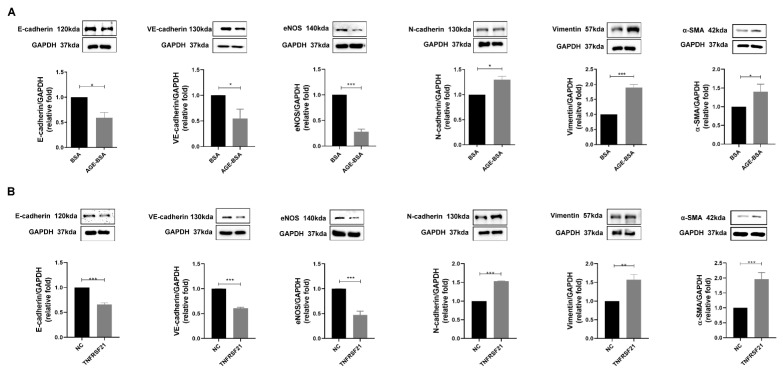
TNFRSF21 (DR6/Fc) promoted EndoMT in CAECs of type 2 DM. (**A**) EndoMT markers, including E-cadherin, VE-cadherin, eNOS, N-cadherin, vimentin, and α-SMA levels, were assessed in HCAECs treated with BSA (300 μg/mL) or AGE-BSA (300 μg/mL) for 48 h (*n* = 3). (**B**) After treatment with TNFRSF21 (10 ng/mL) for 48 h, EndoMT markers were examined in HCAECs using Western blotting. * *p* < 0.05, ** *p* < 0.01, *** *p* < 0.001 by Student’s *t*-test.

EndoMT is one of the major pathophysiologic mechanisms of diabetic heart disease [24,25]. According to the potential impact of TNFRSF21 on left ventricular structure and systolic dysfunction, we investigated whether TNFRSF21 promoted to EndoMT in HCAECs. Both AGEs and TNFRSF21 induced EndoMT process, including decreased expression of endothelial markers such as E-cadherin, VE-cadherin, eNOS and increased expression of mesenchymal markers such as N-cadherin, vimentin, and α-SMA in HCAECs (Figure 4A,B). As per our hypothesis, TNFRSF21 contributed to EndoMT induced by AGEs in CAECs.

### 3.5. TNFRSF21 Increased Permeability of HCAECs

**Figure 5:**
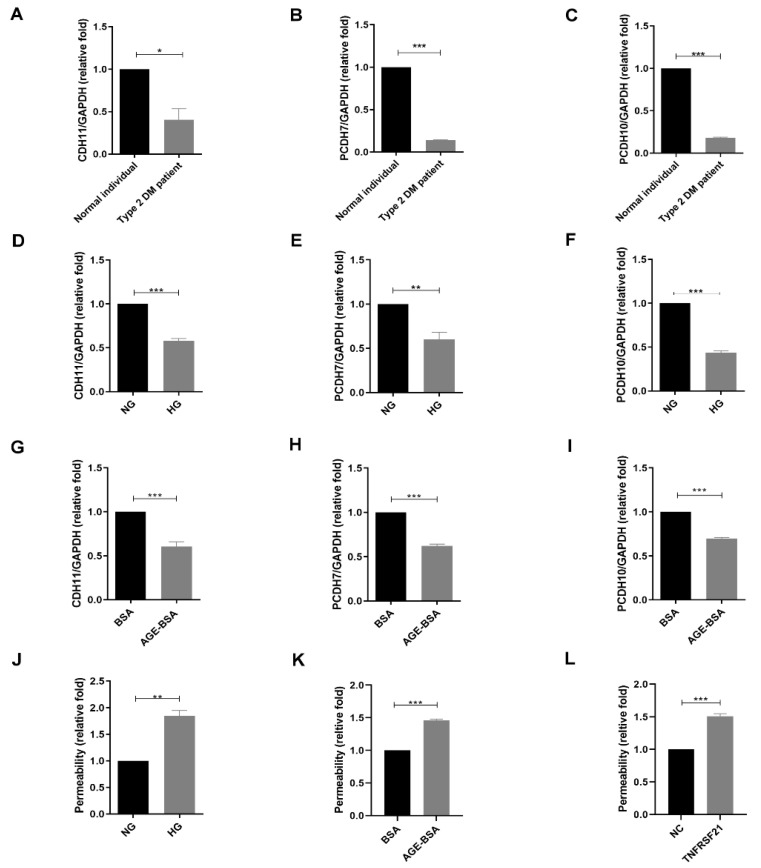
TNFRSF21 induced increased permeability within HCAECs. CDH11, PCDH7 and PCDH11 mRNA were assessed in primary CAECs of normal individual and type 2 DM patient (**A**–**C**), in HCAECs treated with NG (5.5 mM) or HG (25 mM) for 24 h (*n* = 3) (**D**–**F**), and in HCAECs treated with BSA (300 μg/mL) or AGE-BSA (300 μg/mL) for 24 h (*n* = 3) (**G**–**I**). (**J**–**L**) The permeability of HCAECs was examined after treatment with NG, HG, BSA, AGE-BSA, normal control (NC), or TNFRSF21 (10 ng/mL) for 48 h (*n* = 3) using FITC-dextran. * *p* < 0.05, ** *p* < 0.01, *** *p* < 0.001 by Student’s *t*-test.

Differential genes of diabetic CAECs involved in cell adhesion according to bioinformatics results were predicted (Table 3). In several cell junction markers, such as CDH11, PCDH7 and PCDH10, mRNA levels were lower in CAECs in a type 2 DM patient compared with those in a normal individual (Figure 5A–C). Both HG (Figure 5D–F) and AGEs (Figure 5G–I) suppressed CDH11, PCDH7 and PCDH10 expression in HCAECs. In addition, both HG and AGEs enhanced the permeability of HCAECs (Figure 5J,K). Moreover, TNFRSF21 also mimicked the effect of AGEs in the enhancement of permeability in HCAECs (Figure 5L). These results suggested TNFRSF21 might be a mediator of AGEs-induced change in permeability of the coronary vessel.

## 4. Discussion

**Figure 6:**
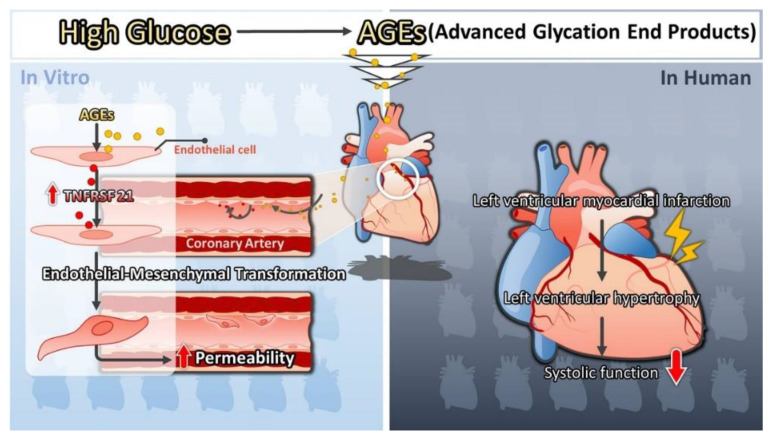
Illustration of the mechanism by which AGEs induced EndoMT and the increase in permeability in CAECs through TNFRSF21 and TNFRSF21 as a biomarker of abnormality of left ventricular structure and systolic function in type 2 DM.

Our study was aimed to explore the signal pathway and novel biomarkers of injury of CAECs in type 2 DM, and further understand the microenvironment changes and mechanisms of diabetic heart disease. Instead of HG, AGEs increased the expression of TNFRSF21 in CAECs, and TNFRSF21 promoted EndoMT in CAECs and further increased the permeability in CAECs. Moreover, elevated levels of serum TNFRSF21 were found in type 2 diabetic patients with LVH than those without LVH. Elevated serum TNFRSF21 levels were correlated with increased LV mass index and with impaired LV systolic function. Our study revealed that TNFRSF21 could be the indicator of changes in subclinical CVD including cardiac structure and function in patients with type 2 DM. This study provided a new insight into understanding the unique mechanism of TNFRSF21 to explain diabetic heart disease development and is identified as a potential biomarker in clinical patients with type 2 DM (Figure 6).

Endothelial dysfunction usually comes before the onset of cardiovascular complications induced by DM [26]. EndoMT has been considered as an important mechanism of endothelial dysfunction, and cardiac fibrosis and hypertrophy, especially in DM [27,28]. Hyperglycemia and consequent AGEs together induce endothelial cells undergoing an imbalance between vasodilation and vasoconstriction as well as the development of vascular complications [26,29]. In addition, hyperglycemia and AGEs could trigger the shift in the endothelium toward the mesenchymal phenotype. Endothelial cells lose their typical cobblestone morphology and tight junctions, degrade the basement membrane, increased motility and the ability to secrete extracellular matrix proteins, and migrate out into the perivascular environment [25,26,30]. Several factors have been known to regulate EndoMT in DM. For example, hyperglycemia can increase oxidative stress, which in turn triggers endothelial cell transformation into myofibroblasts and vascular remodeling [31,32]. In addition, several signaling pathways, such as TGF-β signaling, Notch signaling, fibroblast growth factor/fibroblast growth factor receptor 1 (FGF/FGFR1) signaling, Smad2/3-mediated pathways, and pro-inflammatory signaling cascades have been reported to modulate EndoMT in DM [17,26,33]. Our study used NGS and bioinformatics analysis to find a novel pathophysiological mechanism of coronary artery injury in DM. TNFRSF21 induced EndoMT and increased permeability in CAECs under AGEs, meaning that TNFRSF21 participates in the pathophysiology of DM-induced macrovascular complications. 

TNFRSF21, also known as death receptor 6 (DR6), is a cell surface receptor of the tumor necrosis factor receptor superfamily [34,35], and has been involved in various cellular events, including apoptosis and tumor growth [35,36,37], although its role in endothelial cell injury of CVD in DM has not been explored. We firstly found AGEs, not HG, elevated TNFRS21 expression, and in turn, TNFRSF21 enhanced the increase in permeability within CAECs and further induced EndoMT in CAECs. Furthermore, serum TNFRSF21 level was positively associated with subclinical CVD, including impaired LV systolic function and structure, in clinical patients with type 2 DM. TNFRSF21 has the potential to be a biomarker of subclinical CVD, and it could assist clinical physicians to detect abnormal cardiovascular structure in a more timely manner and function to further prevent the onset or progression of clinical CVD in type 2 DM.

## 5. Conclusions

In conclusion, this study demonstrated the role of TNFRSF21 in coronary artery injury of DM, which could contribute to EndoMT in CAECs and enhance permeability within CAECs. TNFRSF21 could also be a clinical biomarker of subclinical CVD in type 2 DM patients. TNFRSF21 might be a potential therapeutic target for DM-induced CVD.

## Figures and Tables

**Table 1:** The mRNA expression of TNFRSF21 and TNFSF4 in normal and diabetic CAECs.

mRNA	Normal Individual FPKM	Type 2 DM FPKM	Log2 (Fold CHANGE)	*p*-Value
TNFRSF21	72.34	187.28	1.37	<0.001
TNFSF4	37.44	128.09	1.77	<0.001

**Table 2:** The clinical characteristics and cardiac parameters of type 2 DM patients.

	All Type 2 DM Patients *n* = 130	No LVH *n* = 107	LVH *n* = 23	*p*-Value
Age, years	63.0 ± 9.2	62.5 ± 9.0	65.3 ± 9.7	0.17
Sex (male), %	76 (59)	61 (57)	15 (62)	0.47
Smoking, %	32 (25)	28 (26)	4 (17)	0.37
Alcohol, %	22 (17)	18 (17)	4 (17)	0.94
Body mass index, kg/m^2^	25.8 ± 4.6	25.5 ± 4.7	27.4 ± 3.9	0.07
Glycated hemoglobin, %	7.0 (6.5, 8.0)	7.0 (6.6, 7.9)	7.1 (6.3, 8.2)	0.78
Serum creatinine, mg/dL	0.9 ± 0.3	0.9 ± 0.3	1.1 ± 0.5	0.08
Cholesterol, mg/dL	168 ± 37	169 ± 34	163 ± 51	0.63
Triglyceride, mg/dL	120 (85, 170)	116 (81, 168)	134 (96, 250)	0.08
High-density lipoprotein, mg/dL	44 ± 11	45 ± 11	40 ± 10	0.05
Low-density lipoprotein, mg/dL	95 ± 28	97 ± 28	82 ± 26	0.02
Systolic blood pressure, mmHg	138 ± 17	137 ± 16	142 ± 20	0.25
Diastolic blood pressure, mmHg	80 ± 9	80 ± 9	81 ± 11	0.88
Left atrium diameter, cm	3.6 ± 0.7	3.5 ± 0.7	3.9 ± 0.6	0.02
Left atrium diameter/Aortic root diameter	1.1 ± 0.2	1.1 ± 0.3	1.1 ± 0.2	0.92
left ventricular mass index, g/m^2^	90.0 ± 33.2	78.2 ± 20.4	144.8 ± 24.8	<0.001
left ventricular ejection fraction, %	70.6 ± 9.2	70.8 ± 8.7	69.5 ± 11.6	0.54
Left ventricular fraction shortening, %	40.8 ± 7.6	40.9 ± 7.3	40.3 ± 9.0	0.79
E/A ratio	0.8 ± 0.3	0.8 ± 0.3	0.8 ± 0.2	0.67
E/A ratio < 1, *n* (%)	96 (76)	80 (76)	16 (76)	1.00

LVH: left ventricular hypertrophy.

**Table 3:** The biologic process of genes differentially expressed in diabetic CAECs in DAVID database.

Term	Count	%	*p*-Value	Genes
cell adhesion	15	13.5	7.05E-07	POSTN, NRP2, PCDH10, ITGB4, NEDD9, ATP1B1, PCDH17, KITLG, CXCL12, CDH11, ANOS1, CD9, COL8A1, MFGE8, CD44
signal transduction	15	13.5	0.010387	FST, PDE2A, NEDD9, FGF2, APLN, EPS8, KITLG, CXCL12, GPRC5A, TNFSF4, NOSTRIN, CYTL1, PLA2R1, TNFRSF21, CAP2
extracellular matrix organization	11	9.9	3.13E-07	FBN2, POSTN, COL1A2, COL13A1, ITGB4, ABI3BP, BGN, COL8A1, HPSE, FGF2, CD44
angiogenesis	6	5.4	0.011621	NRP2, EMCN, GJA5, COL8A1, ANGPTL4, MFGE8
skeletal system development	5	4.5	0.009732	JAG2, POSTN, COL1A2, GJA5, CDH11
axon guidance	5	4.5	0.016067	SPTBN5, NRP2, CXCL12, ANOS1, SLIT2
response to hypoxia	5	4.5	0.020796	POSTN, CXCL12, PLAT, ANGPTL4, ATP1B1

## Data Availability

The RNA sequencing data generated in this publication have been provided in Supplement Materials.

## Supplementary Materials

The following supporting information can be downloaded at: https://www.mdpi.com/article/10.3390/biomedicines10061282/s1. RNA NGS data. Table S1: Target sequence of the materials utilized in the study.
