# Supplementary material for: Tumor Necrosis Factor Receptor Superfamily Member 21 Induces Endothelial-Mesenchymal Transition in Coronary Artery Endothelium of Type 2 Diabetes Mellitus
Source: Biomedicines. 2022 May 30;10(6):1282. doi: 10.3390/biomedicines10061282 (PMC9220259; doi:10.3390/biomedicines10061282)
Supplement: Supplementary file 1 [file biomedicines-10-01282-s001.zip › biomedicines-1691997-supplementary/Table S1.pdf]

Table S1. Target sequence of materials utilized in the study

gene	Primer	
TNFRSF21	Forward	AGGATCCCAGTGCCATTGTG
	Reverse	CAGCAACCTCCCTCTCACTG
TNFSF4	Forward	CACATCGGTATCCTCGAATTCA
	Reverse	TCCTGGGAGAAGTAGCCCTT
CDH11_	Forward	AGAAATCCACAATCGGCATC
	Reverse	ACAATTGGCTGGTTGGAAAG
PCDH7	Forward	GACTTCGAGGTGTCGGTGAT
	Reverse	GTGGGCAGCAGGTAAAGTGT
PCDH10	Forward	GCGCTCTCCACCAGTAAGTC
	Reverse	GGATCTGGCACTCGAGGATA
GAPDH	Forward	GAGTCAACGGATTTGGTCGT
	Reverse	TTGATTTTGGAGGGATCTCG
